# A presumptive association between obsessive compulsions and asymmetric temporal lobe atrophy: a case report

**DOI:** 10.1186/s13256-021-03228-z

**Published:** 2022-01-20

**Authors:** Thiago Paranhos, Tiago Lucas, Antonio de Salles, Jorge Moll, Ricardo de Oliveira-Souza

**Affiliations:** 1 Cognitive and Behavioral Neuroscience Unit, D’Or Institute for Research & Education (IDOR), Rio de Janeiro, RJ Brazil; 2 School of Medicine, Federal University of Rio de Janeiro, Rio de Janeiro, Brazil; 3 NeuroSapiens and Rede D’Or-São Luiz, São Paulo, São Paulo Brazil; 4 Department of Neurosurgery, University of California, Los Angeles, CA USA; 5 Hospital Universitário Gaffrée e Guinle, Federal University of the State of Rio de Janeiro, Rio de Janeiro, RJ Brazil

**Keywords:** Obsessive–compulsive disorder, Ritualistic behaviors, Acquired compulsions, Temporal variant FTD, Logopenic variant of primary progressive aphasia, Frontotemporal dementia, Case report

## Abstract

**Background:**

The relatively isolated atrophy of the temporal lobes leads to a clinical radiological pattern, referred to as the temporal variant of frontotemporal dementia. While semantic dementia and behavioral variant frontotemporal dementia are classically related to this syndrome, the logopenic variant of primary progressive aphasia has been less commonly reported. This case report aims to give a pictorial description of a case in which a patient with asymmetric temporal lobe atrophy presented with the logopenic variant of primary progressive aphasia and complex rituals of cleanliness.

**Case presentation:**

We report on the case of a 68-year-old, right-handed White woman with complex rituals and progressive speech impairment. The obsessive–compulsive rituals represented an exacerbation of lifelong preoccupations with cleanliness and orderliness that were praised by her relatives. Neuropsychological assessment revealed a striking impairment of language and memory, with relative sparing of tool-use praxis and visuospatial skills. Magnetic resonance imaging and ^18^fluorodeoxyglucose-positron emission tomography scans showed bilateral asymmetrical temporal lobe atrophy and hypometabolism. A year later, she was still able to entertain conversation for a short while, but her vocabulary and fluency had further declined. Praxis and visuospatial skills remained intact. She did not experience pathological elation, delusions, or hallucinations. The disease followed a relentless progression into a partial Klüver–Bucy syndrome, abulia, and terminal dementia. She died from acute myocardial infarction 8 years after the onset of aphasia. The symptoms and their temporal course supported a diagnosis of logopenic variant of primary progressive aphasia due to asymmetric temporal variant frontotemporal lobar degeneration.

**Conclusions:**

This report gives a pictorial description of a temporal variant of frontotemporal dementia in a patient who presented with worsening of a lifelong obsessive–compulsive disorder and logopenic variant of primary progressive aphasia.

**Supplementary Information:**

The online version contains supplementary material available at https://doi.org/10.1186/s13256-021-03228-z.

## Background

Obsessive–compulsive disorder (OCD) has been reported as a consistent, albeit uncommon, manifestation of brain injury [1]. Among the neurodegenerative diseases, an association between OCD and frontotemporal dementia (FTD) has been noted since the first attempts to differentiate FTD from Alzheimer’s disease on clinical and phenomenological grounds [2]. In such cases, OCD emerged as a late-onset disorder [3], as the reemergence [4] or aggravation [5] of a lifelong disorder. To date, the association between OCD and FTD cannot be reliably predicted from the behavioral or neuroimaging patterns of individual cases [6]. These issues have both theoretical and practical implications: on the one hand, they may provide clues to the histopathological substrates in individual cases; on the other hand, they may contribute unique insights into the neurological underpinnings of OCD. Significant strides in this direction have begun to be made. For example, OCD seems to be more common in the semantic variety of primary progressive aphasia [7], while complex compulsions are chiefly related to asymmetric atrophy of the temporal lobes [8]. Unfortunately, most studies do not mention whether OCD already existed as a developmental disorder before the onset of dementia. Moreover, different neural circuits may underpin different dimensions of OCD [9]. Clearly, additional analyses of individual cases and of large series are needed before satisfactory answers can be offered.

The goal of this communication is to contribute a pictorial illustration of a case of progressive aphasia with prominent obsessive–compulsive (OC) symptoms early in the course of the illness. We believe the present case is unique in at least three respects. First, it provides a vivid illustration of a real-life presentation of progressive aphasia with prominent OC symptoms; second, it supports the validity of the rare association between the logopenic variant of primary progressive aphasia (lvPPA) and late-onset OCD; and third, it calls attention to the often-overlooked association between the temporal variant frontotemporal dementia and lifelong OC symptoms, which were mild until the onset of the degenerative process.

## Case presentation

A 68-year-old, right-handed, married White housewife presented with a complaint of failing memory in February 2010. According to her husband, over the previous 3 years his wife’s ability to retrieve the name of familiar objects (for example, scissors, spoon, watch) slowly declined, although she continued to use them normally. At the same time, she found it increasingly difficult to recall the names of friends and relatives that she met only occasionally. Paradoxically, she retained the ability to recall personal facts about people that she failed to recognize or name. For example, she knew that the husband of a friend whom she had not met for a while had recently died; yet she neither recognized her friend nor recalled her name.

Several months before her first appointment, the patient became overly concerned about cleanliness and orderliness. Initially, she swept the sidewalk in front of her house twice a day “to prevent the leaves from clogging the manhole.” In a few weeks, she began to set fire to the piles of leaves and trash that she collected and stacked along the sidewalk of her block several times a day. She would soon be spending most of the day cleaning the street. Attempts to block her way out to the sidewalk increased her level of anxiety and provoked angry protests, which were usually enough for her relatives to let her out. On one occasion, she got into the yard of a nearby house without permission and set fire to a pile of trash that the neighbor had collected the day before. At a friend’s house, she swept the leaves, cleaned the garden flowers, and tidied up the terrace. At her granddaughter’s first birthday party, she left the guests and went to sweep her daughter’s mother-in-law’s backyard. She also continuously searched for plastic bags, empty bottles, and small objects along the way, which she would pick up and bring home, finally throwing them in the trash can. She maintained her regular sleep schedule, with occasional night awakenings; at these times, she would get up to do the laundry. At odds with the patient’s pleasant, albeit reserved, nature, her manners became rude and inadequate. Unless she was closely surveilled, she would pick up food from strangers’ dishes in restaurants and other public places. She abandoned complex routines and hobbies, such as sewing, which she had always been fond of. She was still able to cook and handle cutlery, as well as to dress without help. The patient admitted to feeling sad and distressed but did not meet formal diagnostic criteria for major depression.

Since an early age, the patient strived to keep everything neat and clean, but tidiness was a chief concern. Everything had to be in the “right” place. A blanket on the couch had to be stretched out, and if anyone had a seat, she hurried to straighten up the blanket as soon as the person got up. However, she never missed appointments or got into trouble because of her concern with cleanliness and tidiness, which her relatives saw as a personal quality.

The patient was born in a small town in the northern part of the state of Rio de Janeiro. Although she came from a low-class family, she got married early in life and soon became pregnant with her son and daughter. Therefore, she spent her life taking care of her home and family. There was no history of hypertension, diabetes, tobacco smoking, or drinking. She had elevated serum cholesterol levels with normal triglycerides. Her mother left the family when she was 7 years old. Her father suffered from persecutory delusions since early adulthood. One brother died at age 60 with a diagnosis of alcoholism. Her only medication was simvastatin 20 mg at bedtime.

On her first appointment, her blood pressure was 135/83 mmHg, and her heart rate was regular at 60 beats per minute; her body temperature was likewise normal (36.7 °C). No abnormalities were found in the general examination. She was oriented to time and place and scored on the lower normal range on the Mini-Mental State Exam (26/30). Spontaneous speech was characterized by a slow rate owing to word-finding pauses, but there was no frank agrammatism. She could read and write, and draw a clock from memory with ease. She missed one out of five serial subtractions of 7 from 100. She could not name everyday objects (scissors, pen, cell phone), but correctly described and pantomimed their use and used them appropriately. She was unable to recall the name and recognize close relatives and friends but retained the ability to recall personal facts about them. She often interrupted the interview and spent several minutes eyeing up the drawers of the room cabinet. She often stopped talking to tidy up the books and documents on the examiner’s desk. She sat, stood up, and walked normally, but the associated movements of the arms were reduced in amplitude, especially on the right; rigidity, tremor, or fasciculations were not seen. Her balance was normal, both when she stood and walked; the pull test elicited normal righting reflexes. There was no Romberg sign.

Neuropsychological assessment (Additional file 1) revealed a striking dissociation between the impairment of language and memory and the preservation of tool-use praxis and visuospatial and visuoconstructional skills; tests for visuospatial neglect were likewise normal (Table 1). Naming was far more compromised than aural and written comprehension, as shown by her performance on the 15-object test (Additional file 1) and on the Multilingual Aphasia Examination [10]. Her memory disorder was due to an impairment of both encoding and recall.

**Table 1:** Neuropsychological assessment

Test or inventory	2010	Range	Normal^a^
Clinical dementia rating	**0.5**	0–5	0
Behav^b^	**2**	0–3	0
Lang	**1.5**	0–3	0
Mini-Mental State Exam			
Total score	**26**	0–30	≥ 26
Temporal orientation	5	0–5	≥ 4
Geographic orientation	5	0–5	≥ 4
Registration	3	0–3	3
Attention and calculation	4	0–5	≥ 4
Recall	**0**	0–3	3
Naming	2	0–2	2
Repetition	1	0–1	1
Execution of written command	1	0–1	1
Sentence writing	1	0–1	1
Copy design	1	0–1	1
Three-stage command	3	0–3	3
Visual naming (15 objects)	**8**	0–15	≥ 14
Multilingual aphasia examination			
Visual naming	**20**	0–60	
Aural comprehension	14	0–18	
Reading comprehension	14	0–18	
Pyramids and palm trees	**34**	0–52	≥ 46
Wisconsin Card Sorting Test			
Categories completed	04	0–6	≥ 4
Perseverative errors	22	0–127	≤ 65
Common (nonperseverative) errors	21	0–128	≤ 33
Total errors	43	0–128	≤ 85
Set failures	02	0–21	≤ 02
3D block construction			
Total score	28	0–29	≥ 24
Model I	06	0–6	06
Model II	08	0–8	≥ 05
Model III	14	0–15	≥ 11
Total time (in seconds)	178	0–∞	≤ 475
Enhanced cued recall			
Immediate recall			
Total (free + cued)	28	0–48	48
Free	15	0–48	≥ 29
Cued	13	0–48	≤ 05
Delayed recall (45 minutes)			
Total (free + cued)	**9**	0–16	16
Free	**1**	0–16	≥ 10
Cued	**8**	0–16	≤ 06
Right–left orientation			
Own body	12	0–12	≥ 11
Examiner’s body	07	0–8	≥ 7
Line bisection L^c^	0.03	0–100	Between −10 and 7
Line bisection C	−0.05	0–100	Between −6 and 8
Line bisection R	0.03	0–100	Between −7 and 12

See Additional file 1 for description of the neuropsychological assessment.

Abnormal results are in **bold** type.

^a^Normative data computed from the idor Normative Data Bank using the *N* = 1 statistics [11]

^b^behav and lang correspond, respectively, to the added domains of behavior and language to the standard Clinical Dementia Rating [12]

^c^Scores on the Line Bisection Test were computed as the percent deviation of lines on the left, center, and right third of the page. Each third contains six lines with lengths ranging from 10 to 20 cm [13]

A year and a half later, the patient had become abulic, and her cognitive impairment had evolved into overt dementia (Mini-Mental State Exam 17/30). She intermittently emerged from the abulic state with attempts to go out and clean the street. Despite the progression to dementia, she remained oriented even to new and unfamiliar places; likewise, she remained able to copy two intersecting pentagons and draw a clock from memory as accurately as she did on her first appointment.

A few trials of 2–3 months of serotonin followed by paroxetin associated with olanzapine early in the course of dementia were of no avail and were suspended. In her final years, her memory impairment, the difficulty in time and place orientation, and the recognition of familiar faces further declined, along with a steady decrease of the compulsions to clean and tidy up. Her cognitive status remained unchanged after a 6-month trial of rivastigmine 12 mg. She developed hyperorality, bringing to mouth inedible objects such as vinegar, liquid soap, skin lotions, and shaving cream. She developed urinary and fecal incontinence in the last 2 years of life. In her final months of life, she was severely abulic and her verbal output was reduced to just a few words. She passed away from acute myocardial infarction in November 2016, with an estimated length of disease of 8 years.

## Neuroimaging findings

**Fig. 1:**
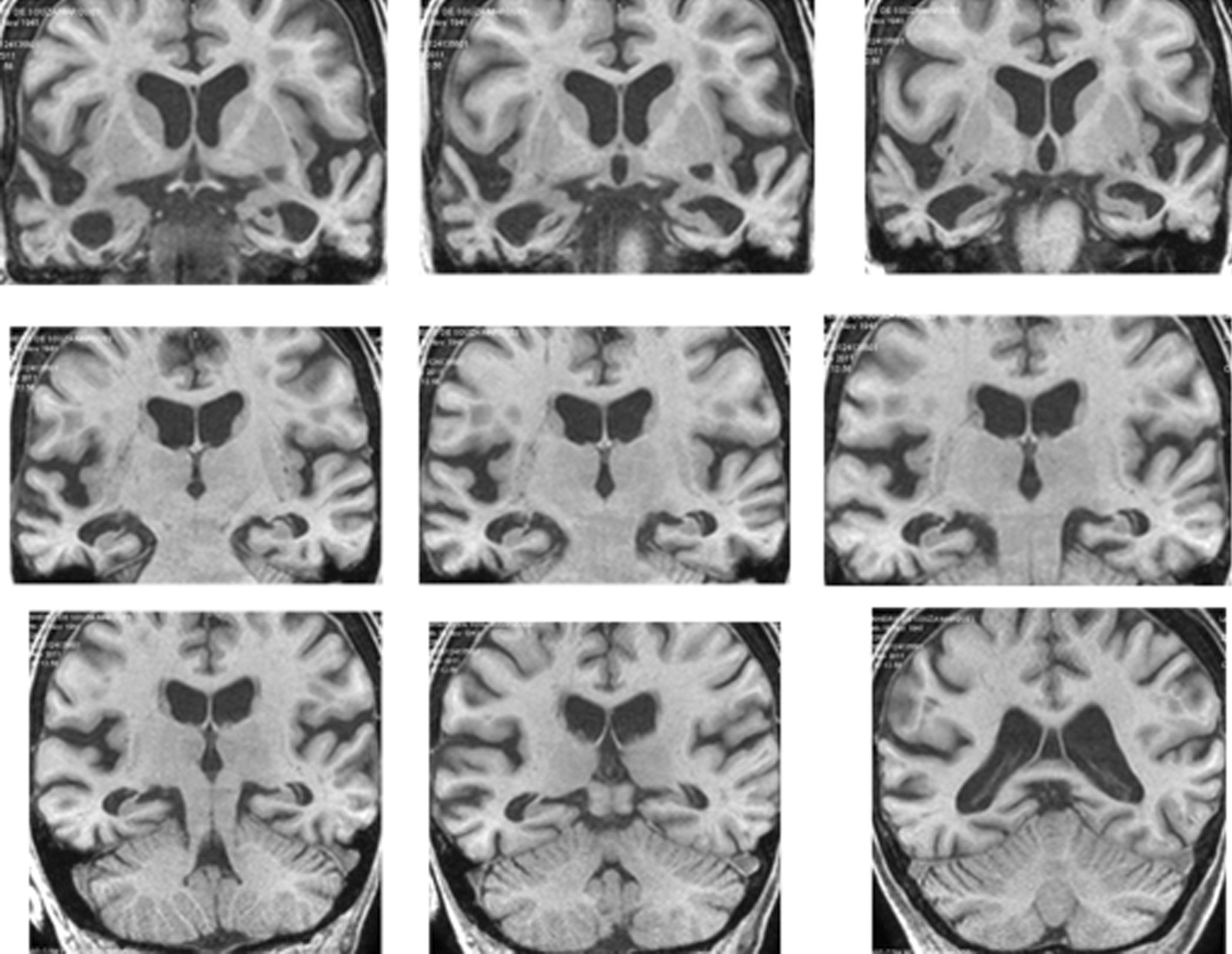
T1-weighted MRI coronal slices through the anterior (upper row), middle (middle row), and posterior (bottom row) third of the cerebral hemispheres. Atrophy is most marked in the temporal lobes, more so on the right, as indicated by the wider Sylvian fissure and temporal horn on this side. The lateral ventricles are also symmetrically enlarged

The patient underwent anatomic and spectroscopic magnetic resonance imaging (MRI) in March 2010 and August 2011 (Fig. 1). The neuroimaging protocols are detailed in Additional file 1. MRI showed supratentorial ventricular ectasia. The right hemisphere was smaller than the left, especially the temporal lobes, as shown by marked enlargement of the temporal horns. The lateral ventricles were also enlarged. Although the head of the caudate nuclei maintained their convex shape, the bicaudate index (0.17) indicated that these nuclei were atrophic independently of whole brain atrophy (detailed in Additional file 1). Scattered hyperintensities, probably corresponding to microangiopathic gliosis, were seen in the periventricular and subcortical white matter on T2 and fluid-attenuated inversion recovery (FLAIR). Their overall volume was of small magnitude; moreover, they were not localized to strategic sites that are known to produce vascular dementia [14]. Perivascular spaces were bilaterally present in the internal capsule and basal ganglia, but there was no evidence of recent ischemic lesions. The left cerebellar hemisphere was slightly smaller than the right, suggestive of crossed cerebellar atrophy. The right upper ventral brainstem was smaller than the left, probably due to a shrinkage of the temporopontine tract. Magnetic resonance spectroscopy revealed a reduction of NAA/Cr (1.35) and an increase of MI/NAA (0.50) in the posteromedial cortices; a year and a half later, these changes were more pronounced: 1.24 and 0.57, respectively.

**Fig. 2:**
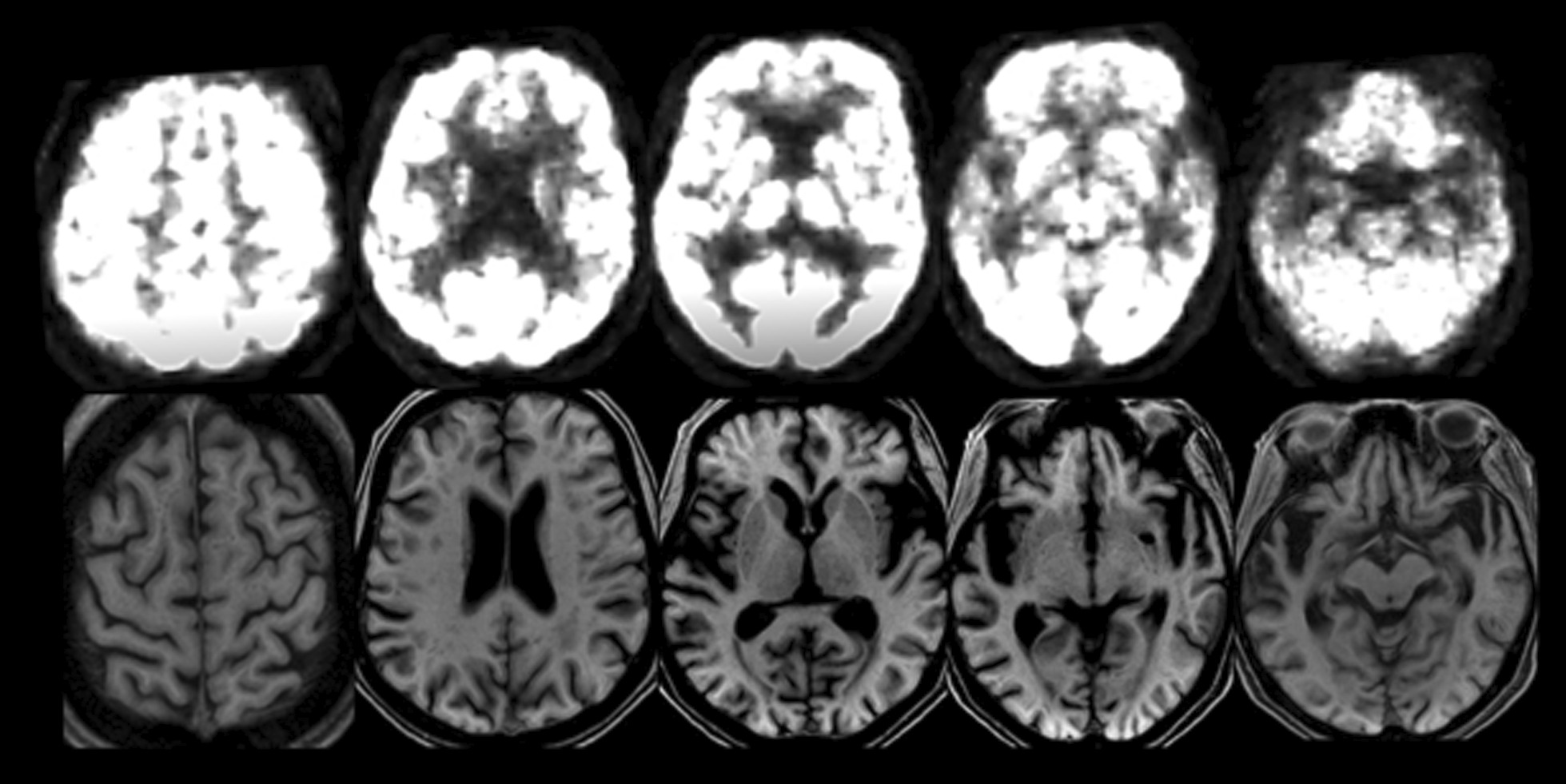
Upper row: ^18^FDG-PET scan showing bilateral hypometabolism in the anterior temporal cortex. The metabolism of the orbitofrontal cortex as well as the caudate nucleus and thalamus is bilaterally normal. Lower row: T1-weighted MRI showing severe bilateral anterior temporal lobe atrophy with relative sparing of the frontal, parietal, and occipital lobes

**Fig. 3:**
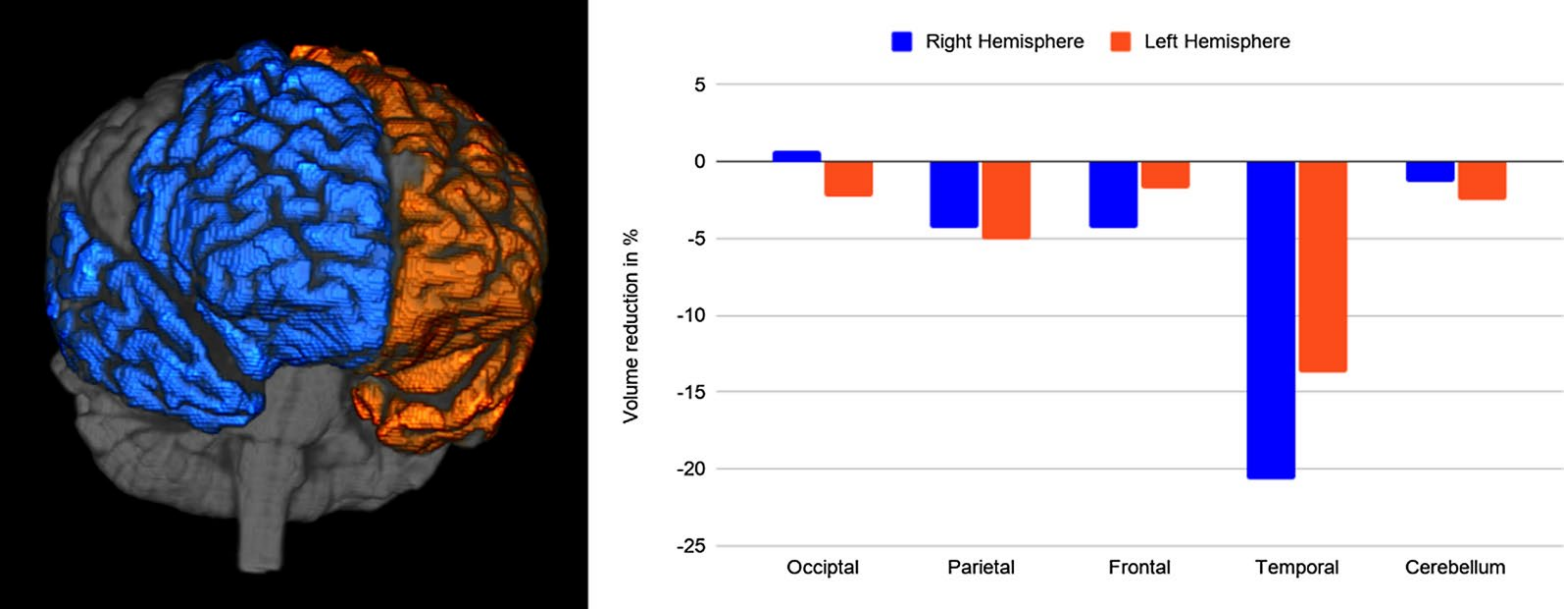
The regions of interest (ROIs) of the frontal and temporal lobes used in the volumetric comparison are displayed on the left. On the right, the reduction of cortical grey matter over a 1-year time span is displayed in a bar chart. The *x* axis represents the analyzed cerebral regions, while the *y* axis represents the percentage volume loss in the right (blue bar) and left (orange bar) hemispheres

A follow-up MRI showed accentuation of the cortical sulci, most markedly in the temporal lobes. An ^18^FDG-PET scan at the time revealed hypometabolism in the anterior temporal lobes. The metabolism of the thalamus and lenticular nucleus, as well as of the frontal, parietal, and occipital cortices was bilaterally normal (Fig. 2). Volumetric analysis displayed global cortical grey matter volumetric reduction over a year, which was more pronounced in the right temporal lobe (Additional file 1) (Fig. 3).

## Discussion

We herein present the case of a middle-aged woman, in whom the worsening of a lifelong pattern of cleanliness preoccupation exposed the presence of a severe neurodegenerative condition. Although it might be argued that a primary psychiatric disorder could explain the behavioral symptoms, evidence of asymmetric temporal lobe atrophy and volumetric cortical shrinkage over a 1-year follow-up points to a neurodegenerative process. We lacked neuroimaging before the clinical presentation, but the temporal atrophy was unlikely to be congenital or present for many years before the first symptoms were noted. Given this case’s clinical course and neuroimaging features, a presumptive diagnosis of lvPPA with OCD due to asymmetric temporal variant FTD was made.

The first 3 years of disease were marked by anomia and word-finding difficulties, episodic memory impairment, and prosopagnosia. A few months before the first appointment, complex rituals of cleanliness became prominent. At the time of presentation, OCD and language impairment were the most distressing symptoms. Besides a great difficulty in naming familiar objects and people, her speech output was hesitating, with frequent word-finding pauses and phonemic substitutions, but no frank agrammatism was observed; in contrast, her comprehension of words and knowledge of object use were, to a great extent, spared. The pattern of language impairment was thus consistent with lvPPA. This diagnosis might be challenged by recent guidelines because repetition, one of the two core criteria for lvPPA, was largely spared in our patient [15]. However, it has also been suggested that such criteria ought to be redefined to include logopenic patients without repetition impairment [16]. It was also intriguing that the right temporal lobe was the region in which atrophy was most severe, and the region that underwent the greatest volumetric reduction over the course of 1 year. While we could speculate that this right-handed patient suffered a “crossed” PPA because of predominant right temporal lobe atrophy [17], PET scan also revealed hypometabolism in the temporal lobe of the left hemisphere.

Neuropsychological assessment revealed impairment of confrontation naming (visual naming subtest of the Multilingual Aphasia Examination; 15-object naming test) and nonverbal object knowledge (pyramids and palm trees). However, word comprehension (aural comprehension subtest of the Multilingual Aphasia Examination) and knowledge of object use evaluated in our first encounter made the diagnosis of semantic variant PPA less likely. The pattern of predominant right temporal lobe atrophy might explain the relative preservation of semantic knowledge, but as the neuropathological process ultimately impinged upon both hemispheres, semantic impairment also became evident [18].

At the time of presentation, OCD was a prominent symptom but was not accompanied by other behavioral abnormalities typical of behavioral variant frontotemporal dementia [19], making this diagnosis less appropriate. Months after the initial presentation, behavioral disturbances became more pronounced, and the syndromes of PPA and behavioral variant frontotemporal dementia coexisted. However, at least for a few months preceding the first appointment, the language impairment and pure OCD were present in relative isolation.

Her husband reported that the patient suffered from low-grade OCD since at least early adulthood. Unfortunately, many reports on late-life OCD do not state whether the compulsions were of recent onset or had been present since early adulthood or even before. The reports that provide this information indicate that the association of OCD and FTD may assume the form of (i) a rekindling or worsening of a lifelong OCD, as was the case of our patient and a few others reported in the literature [4] or (ii) a true onset of OC symptoms and kindred repetitive behaviors in patients without evidence of premorbid OCD [20].

Our patient’s OC symptoms represented a worsening of lifelong rituals of tidiness, which came to the forefront of the clinical manifestations roughly in parallel with the onset of speech difficulties. The onset and worsening of aphasia seemed to occur in tandem with the aggravation of her lifelong OC rituals over the first two-thirds of the course of the disease. This association steadily faded away as dementia and abulia eventually dominated the clinical picture. By that time, and in keeping with the bilateral anterior temporal damage, she manifested the core symptoms of Klüver–Bucy syndrome [21].

Few studies have described focal brain injuries that either cause or cure OCD [22]; functional imaging investigations have also led to conflicting results because the same structures that appear hyperactive in some studies may appear hypoactive in others [23]. However, the dramatic amelioration of OCD by the surgical interruption of connections between the orbitofrontal cortex and medial thalamus, as well as the results of cingulotomy [24], indicate that the net pathophysiological effect is hyperactivity at some point within frontostriatal and anterior cingulate circuits [25]. In principle, the same degenerative processes that caused the cognitive symptoms of FTD might also have been responsible for the emergence, reemergence, or worsening of stereotypies and compulsions. If these phenomena are related, as their clinical parallelism suggests, then the cognitive and OC rituals might represent, respectively, symptoms of deficit and release caused by a single underlying pathological process [26]. In due time, the progress of pathology ultimately impinged on the relatively intact neural circuits that mediate the unrestrained OC symptoms, and they disappeared altogether. This course is consistent with the disease progression of our patient, in whom the relative sparing of the anterior cingulate, orbitofrontal cortices, and striothalamic metabolism were in sharp contrast with the atrophy and hypometabolism of the temporal lobes.

It is likely that the hypofunctional temporal lobes released critical anterior cingulate and orbitostriatal circuits responsible for the OC symptoms that were praised by her relatives until the degenerative process gained momentum. This hypothesis underscores a modulating role of the anterior temporal lobes on OC symptoms, an idea that finds support in the emergence of severe OCD in patients undergoing temporal lobectomy for the treatment of medically refractory seizures [27], and in recent research that found that complex compulsions such as those presented by our patient were related to asymmetric atrophy of the temporal lobes in 90 cases of FTD [8]. In contrast, simple repetitive motor and verbal actions seem to be primarily related to caudate atrophy [28].

The lack of histopathologic examination precluded the establishment of a final diagnosis in our case. The lvPPA is more commonly associated with Alzheimer’s pathology [29], which might explain the difficulty to differentiate Alzheimer’s disease from lvPPA in certain cases [30]. Given the similarity of our case to the pattern of semantic dementia from right temporal variant frontotemporal dementia (tvFTD), TDP-43 pathology should also be considered [31]. To complicate the issue even further, the caudate atrophy seen on MRI is more commonly associated with frontotemporal lobar degeneration fused-in-sarcoma (FTLD-FUS) pathology [32].

Our case adds to the rapidly growing registry of classically described psychopathological syndromes that have been reproduced by cases of FTD [33]. The evaluation of the large numbers of patients searching for diagnosis and treatment of acquired psychopathological syndromes has reawakened the interest in neuropsychiatry, and contributed to prove the fallacy of segregating the *practice* of neurology and psychiatry from each other. This renewed interest, which has shed new light over the neuroanatomic and neurochemical underpinnings of psychopathology, has opened novel surgical and pharmacological therapeutic opportunities to investigate the neural underpinnings of classical psychopathological syndromes. A deeper understanding of the involved pathways is already encouraging the development of new neuromodulation targets to help patients with neurodegenerative diseases [34, 35]. At the same time, they have opened new avenues for research on the neurochemical correlates of both developmental and acquired neuropsychiatric disorders.

## Conclusion

We report the case of a woman with lvPPA and OCD due to tvFTD. The complex rituals of cleanliness were the main symptoms that motivated the patient’s husband to seek medical attention. This report highlights the importance of a thorough neuropsychiatric investigation of patients that present to consultation with a late-onset psychiatric syndrome.

## Supplementary Information

**Additional file 1.** Neuropsychological battery and neuroimaging protocols.

## Data Availability

Data sharing is not applicable to this article as no datasets were generated or analysed during the current study.

## Abbreviations

bvFTD: Behavioral variant frontotemporal dementia
FTD: Frontotemporal dementia
lvPPA: Logopenic variant of primary progressive aphasia
MRI: Magnetic resonance imaging
OCD: Obsessive–compulsive disorder
tvFTD: Temporal variant frontotemporal dementia

## References

[1] Fontenelle L. The clinical neuropsychiatry of “organic” obsessive–compulsive disorder. In: Fontenelle L, Yücel M, editors. A transdiagnostic approach to obsessions, compulsions and related phenomena. Cambridge: Cambridge University Press; 2019. p. 383–400.

[2] Miller BL, Darby AL, Swartz JR, Yener GG, Mena I. Dietary changes, compulsions and sexual behavior in frontotemporal degeneration. Dementia. 1995;6:195–9. 10.1159/000106946 7550598

[3] Bersano E, Sarnelli MF, Solara V, De Marchi F, Sacchetti GM, Stecco A, Corrado L, D’Alfonso S, Cantello R, Mazzini L. A case of late-onset OCD developing PLS and FTD. Amyotroph Lateral Scler Frontotemporal Degener. 2018;19:463–5. 10.1080/21678421.2018.1440405 29451027

[4] Dondé C, Lepetit A, Dorey JM, Herrmann M. Late-life atypical reactivation of obsessive–compulsive disorder associated with frontotemporal dementia. Rev Neurol (Paris). 2019;175:205–6. 10.1016/j.neurol.2018.04.015 30665687

[5] Karnik NS, D’Apuzzo M, Greicius M. Non-fluent progressive aphasia, depression, and OCD in a woman with progressive supranuclear palsy: neuroanatomical and neuropathological correlations. Neurocase. 2006;12:332–8. 10.1080/13554790601125957 17182396

[6] Rauch SL, Dougherty DD, Shin LM, Albert N, Manzo P, Leahy L, Fischman AJ, Jenike MA, Baer L. Neural correlates of factor-analyzed OCD symptom dimensions: a PET study. CNS Spectr. 1998;3:37–43.

[7] Mitchell E, Tavares TP, Palaniyappan L, Finger EC. Hoarding and obsessive compulsive behaviours in frontotemporal dementia: clinical and neuroanatomic associations. Cortex. 2019;121:443–53. 10.1016/j.cortex.2019.09.012 31715541

[8] Rosso SM, Roks G, Stevens M, de Koning I, Tanghe HLJ, Kamphorst W, Ravid R, Niermeijer MF, van Swieten JC. Complex compulsive behaviour in the temporal variant of frontotemporal dementia. J Neurol. 2001;248:965–70. 10.1007/s004150170049 11757960

[9] van den Heuvel OA, Remijnse PL, Mataix-Cols D, Vrenken H, Groenewegen HJ, Uylings HBM, Uylings HBM, van Balkom AJLM, Veltman DJ. The major symptom dimensions of obsessive–compulsive disorder are mediated by partially distinct neural systems. Brain. 2009;132:853–68. 10.1093/brain/awn267 18952675

[10] Benton AL, Hamsher KS, Sivan AB. Multilingual aphasia examination. 3rd ed. Lutz: Psychological Assessment Resources; 1994.

[11] Crawford JR, Howell DC, Garthwaite PH. Payne and Jones revisited: estimating the abnormality of test score differences using a modified paired samples t test. J Clin Exp Neuropsychol. 1998;20:898–905. 10.1076/jcen.20.6.898.1112 10484700

[12] Knopman S, Weintraub S, Pankratz VS. Language and behavior domains enhance the value of the clinical dementia rating scale. Alzheimer’s Dement. 2011;7:293–9. 10.1016/j.jalz.2010.12.006 PMC3096831 21575870

[13] Schenkenberg T, Bradford DC, Ajax ET. Line bisection and unilateral visual neglect in patients with neurologic impairment. Neurology. 1980;30:509–17. 10.1212/wnl.30.5.509 7189256

[14] Tatemichi TK, Desmond DW, Prohovnik I. Strategic infarcts in vascular dementia. A clinical and brain imaging experience. Arzneimittelforschung. 1995;45:371–85. 7763329

[15] Gorno-Tempini ML, Hillis AE, Weintraub S, Kertesz A, Mendez M, Cappa SF, Ogar JM, Rohrer JD, Black S, Boeve BF, Manes F, Dronkers NF, Vandenberghe R, Rascovsky K, Patterson K, Miller BL, Knopman DS, Hodges JR, Mesulam MM, Grossman M. Classification of primary progressive aphasia and its variants. Neurology. 2011;76:1006–14. 10.1212/WNL.0b013e31821103e6 PMC3059138 21325651

[16] Mesulam MM, Weintraub S. Is it time to revisit the classification guidelines for primary progressive aphasia? Neurology. 2014;82:1108–9. 10.1212/WNL.0000000000000272 24598706

[17] Demirtas-Tatlidede A, Gurvit H, Oktem-Tanor O, Emre M. Crossed aphasia in a dextral patient with logopenic/phonological variant of primary progressive aphasia. Alzheimer Dis Assoc Disord. 2012;26:282–4. 10.1097/WAD.0b013e31823346c6 21959362

[18] Seeley WW, Bauer AM, Miller BL, Gorno-Tempini ML, Kramer JH, Weiner M, Rosen HJ. The natural history of temporal variant frontotemporal dementia. Neurology. 2005;64:1384–90. 10.1212/01.WNL.0000158425.46019.5C PMC2376750 15851728

[19] Rascovsky K, Hodges JR, Knopman D, Mendez MF, Kramer JH, Neuhaus J, van Swieten JC, Seelaar H, Dopper EG, Onyike CU, Hillis AE, Josephs KA, Boeve BF, Kertesz A, Seeley WW, Rankin KP, Johnson JK, Gorno-Tempini ML, Rosen H, Prioleau-Latham CE, Lee A, Kipps CM, Lillo P, Piguet O, Rohrer JD, Rossor MN, Warren JD, Fox NC, Galasko D, Salmon DP, Black SE, Mesulam M, Weintraub S, Dickerson BC, Diehl-Schmid J, Pasquier F, Deramecourt V, Lebert F, Pijnenburg Y, Chow TW, Manes F, Grafman J, Cappa SF, Freedman M, Grossman M, Miller BL. Sensitivity of revised diagnostic criteria for the behavioural variant of frontotemporal dementia. Brain. 2011;134:2456–77. 10.1093/brain/awr179 PMC3170532 21810890

[20] Ames D, Cummings JL, Wirshing WC, Quinn B, Mahler M. Repetitive and compulsive behavior in frontal lobe degenerations. J Neuropsychiatry Clin Neurosci. 1994;6:100–13. 10.1176/jnp.6.2.100 8044031

[21] Nahm FKD. Heinrich Klüver and the temporal lobe syndrome. J Hist Neurosci. 1997;6:193–208. 10.1080/09647049709525702 11619521

[22] Berthier ML, Kulisevsky J, Gironell A, Heras JA. Obsessive–compulsive disorder associated with brain lesions: clinical phenomenology, cognitive function, and anatomic correlates. Neurology. 1996;47:353–61. 10.1212/wnl.47.2.353 8757004

[23] Menzies L, Chamberlain SR, Laird AR, Thelen SM, Sahakian BJ, Bullmore ET. Integrating evidence from neuroimaging and neuropsychological studies of obsessive–compulsive disorder: the orbitofrontal-striatal model revisited. Neurosci Biobehav Rev. 2008;32:525–49. 10.1016/j.neubiorev.2007.09.005 PMC2889493 18061263

[24] Jenike MA, Bear L, Ballantine HT, Martuza RL, Tynes S, Giriunas I, Buttolph ML, Cassem NH. Cingulotomy for refractory obsessive–compulsive disorder: a long term follow up of thirty-three patients. Arch Gen Psychiatry. 1991;48:548–55. 10.1001/archpsyc.1991.01810300060009 2039338

[25] Matinez-Alvarez R. Ablative surgery for obsessive–compulsive disorder. In: Sun B, De Salles A, editors. Neurosurgical treatments for psychiatric disorders. Springer; 2015. p. 105–12.

[26] Berrios GE. Positive and negative symptoms and Jackson. A conceptual history. Arch Gen Psychiatry. 1985;42:95–7. 10.1001/archpsyc.1985.01790240097011 3881095

[27] Kulaksizoglu IB, Bebek N, Baykan B, Imer M, Gürses C, Sencer S, Oktem-Tanör O, Gökyigit A. Obsessive–compulsive disorder after epilepsy surgery. Epilepsy Behav. 2004;5:113–8. 10.1016/j.yebeh.2003.11.015 14751216

[28] Maia ASSF, Barbosa ER, Menezes PR, Miguel EC. Relationship between obsessive compulsive disorders and diseases affecting primarily the basal ganglia. Revista do Hospital das Clínicas. 1999;54:213–21. 10.1590/s0041-87811999000600008 10881070

[29] Mesulam MM, Weintraub S, Rogalski EJ, Wieneke C, Geula C, Bigio EH. Asymmetry and heterogeneity of Alzheimer’s and frontotemporal pathology in primary progressive aphasia. Brain. 2014;137:1176–92. 10.1093/brain/awu024 PMC3959558 24574501

[30] Beber BC, Kochhann R, da Silva BM, Chaves MLF. Logopenic aphasia or Alzheimer’s disease: different phases of the same disease? Dement Neuropsychol. 2014;8:302–7. 10.1590/S1980-57642014DN83000016 PMC5619409 29213918

[31] Josephs KA, Whitwell JL, Knopman DS, Boeve BF, Vemuri P, Senjem ML, Parisi JE, Ivnik RJ, Dickson DW, Petersen RC, Jack CR Jr. Two distinct subtypes of right temporal variant frontotemporal dementia. Neurology. 2009;73:1443–50. 10.1212/WNL.0b013e3181bf9945 PMC2779005 19884571

[32] Josephs KA, Whitwell JL, Parisi JE, Peterson RC, Boeve BF, Jack CR Jr, Dickson DW. Caudate atrophy on MRI is a characteristic feature of FTLD-FUS. Eur J Neurol. 2010;17:969–75. 10.1111/j.1468-1331.2010.02975.x PMC2989679 20236174

[33] Lanata SC, Miller BL. The behavioural variant frontotemporal dementia (bvFTD) syndrome in psychiatry. J Neurol Neurosurg Psychiatry. 2016;87:501–11. 10.1136/jnnp-2015-310697 PMC4755931 26216940

[34] McKinnon C, Gros P, Lee DJ, Hamani C, Lozano AM, Kalia LV, Suneil KK. Deep brain stimulation: potential for neuroprotection. Ann Clin Transl Neurol. 2018;6:174–85. 10.1002/acn3.682 PMC6331208 30656196

[35] Pereira JLP, Downes A, Gorgulho A, Patel V, Malkasia D, De Salles A. Alzheimer’s disease: the role for neurosurgery. Surg Neurol Int. 2014;5:385–90. 10.4103/2152-7806.140191 PMC4173304 25289167

