# Supplementary material for: A presumptive association between obsessive compulsions and asymmetric temporal lobe atrophy: a case report
Source: J Med Case Rep. 2022 Jan 20;16:21. doi: 10.1186/s13256-021-03228-z (PMC8772087; doi:10.1186/s13256-021-03228-z)
Supplement: Supplementary file 1 — Additional file 1. Neuropsychological battery and neuroimaging protocols. [file 13256_2021_3228_MOESM1_ESM.docx]

**Additional Files**

**Obsessive Compulsions due to Asymmetric Temporal Lobe Atrophy: A Case Report**

**Additional Figure 1:** Bifrontal and Bicaudate Ratios (Aylward et al., 1991)


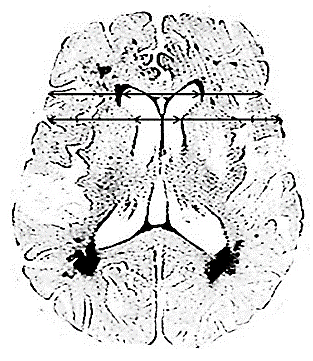
*Bifrontal Ratio (BFR)*: The BFR is a measure of ventricular enlargement independent of caudate atrophy. It is measured on the axial section in which the frontal horns are clearly visible and the septum is the thinnest. The BFR is the distance between the most lateral tips of the frontal horns divided by brain width along the same line.

*Bicaudate Ratio (BCR)*: The BCR is a measure of caudate atrophy independent of whole brain atrophy. It is measured on the same axial section as the BFR. The BCR is the minimal distance between the caudate indentations of the frontal horns divided by brain width along the same line.

Bicaudate and bifrontal ratios were measured for our patient and seven age-matched healthy controls based on T1-weighted imaging. The patient’s ratios from 2010 and 2011 T1 sequences were individually compared to the normative data by means of N = 1 statistics (Crawford et al., 1998). The comparison did not reach statistical significance for the 2010 sequence. In turn, BCR and BFR from 2011 were statistically higher than the normative data.

**Neuropsychological assessment**


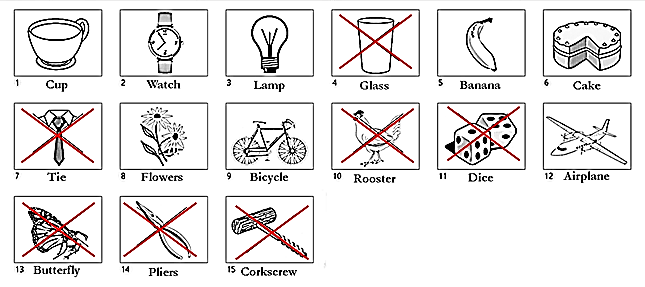
The patient underwent a neuropsychological evaluation to assess global cognition (Brucki et al., 2003), naming, aural and reading comprehension (Benton et al., 1994a), semantic knowledge (Howard et al., 1992), right-left orientation (Benton et al., 1994b), memory encoding and retrieval, both immediate and after a 45-minute delay (Grober et al., 1988), three dimensional block construction (Benton et al., 1994b), concept formation and executive flexibility (Anderson et al., 1991), and visuospatial attention (Schenkenberg et al., 1980). Naming was also assessed with the 15-Object Naming Test (Additional Figure 2). Executive production and visuospatial attention were also assessed. Finally, she was asked to draw a clock from memory and set the hands to twenty to three. The ability to draw clocks is a quick and reliable test for the elicitation of impairments of planning and visuospatial neglect (Mendez et al., 1992).

**Additional Figure 2: 15-Object Naming Test.** The items that the patient missed are cancelled (X).

**Neuroimaging**

The patient underwent evaluations with a 3.0 T magnetic resonance imaging (MRI) Siemens Somatom scanner in March 2010 and in August 2011. Magnetic resonance spectroscopy was also performed. The progression of lobar atrophy over a year span was assessed with the FreeSurfer software package (Dale et al., 1999; Fischl et al., 2004). T1 volumes obtained in 2010 and 2011 were pre-processed and parcellated in the cerebral cortical regions and cerebellum according to the Desikan-Killiany atlas (Desikan et al., 2006). The volumes of each of the following regions of each hemisphere were calculated for the 2010 and 2011 scans: the frontal, temporal, parietal and occipital lobes, and the right and left cerebellar hemispheres. The percentage of cortical grey matter reduction for each lobe was plotted in a bar chart. In August 2011, a ^18^FDG-PET was also obtained.

**References**

Anderson SW, Damasio H, Jones RD, Tranel D. Wisconsin Card Sorting Test performance as a measure of frontal lobe damage*.* J Clin Exp Neuropsychol. 1991; 13:909-922.

Aylward EH, Schwartz J, Machlin S, Pearlson G. Bicaudate ratio as a measure of caudate volume on MR images. American Journal of Neuroradiology. 1991;12:1217-1222.

Benton AL, Hamsher KS, Sivan AB. Multilingual Aphasia Examination. 3^rd^ ed. Lutz, FL: Psychological Assessment Resources; 1994.

Benton AL, Sivan AB, Hamsher KS. (1994b). Contributions to Neuropsychological Assessment. 2^nd^ ed. New York, New York: Oxford University Press; 1994b.

Brucki SMD, Nitrini R, Caramelli P, Bertolucci PHF, Okamoto IH. Suggestions for utilization of the Mini-Mental State Examination in Brazil. Arq Neuropsiquiatr. 2003;*61:*777-781.

Crawford JR, Howell DC, Garthwaite PH. Payne and Jones revisited: estimating the abnormality of test score differences using a modified paired samples t test. J Clin Exp Neuropsychol. 1998;20:898-905.

Dale AM, Fischl B, Sereno MI. Cortical surface-based analysis I: segmentation and surface reconstruction. Neuroimage. 1999;9:179-194.

Desikan RS, Segonne F, Fischl B, Quinn BT, Dickerson BC, Blacker D, Buckner RL, Dale AM, Maguire RP, Hyman BT, Albert MS, Killiany RJ. An automated labeling system for subdividing the human cerebral cortex on MRI scans into gyral based regions of interest. Neuroimage. 2006;31: 968-980.

Fischl B, van der Kouwe A, Destrieux C, Halgren E, Ségonne F, Salat DH, Busa E, Seidman LJ, Goldstein J, Kennedy D, Caviness V, Makris N, Rosen B, Dale AM. Automatically parcellating the human cerebral cortex. Cerebral Cortex. 2004;14:11-22.

Grober E, Buschke H, Crystal H, Bang S, Dresner R. Screening for dementia by memory testing. Neurology. 1988;38:900-903.

Howard D, Patterson K. The Pyramids and Palm Trees Test: a test of semantic access from pictures and words. Bury St. Edmunds, Suffolk: Thames Valley Test Company; 1992.

Mendez MF, Ala T, Underwood KL. Development of scoring criteria for the clock drawing task in Alzheimer’s disease. J Am Geriatr Soc. 1992;40:1095-1099.

Schenkenberg T, Bradford DC, Ajax ET. Line bisection and unilateral visual neglect in patients with neurologic impairment. Neurology. 1980;30:509-517.
